# The Chemokine CXCL16 and Its Receptor, CXCR6, as Markers and Promoters of Inflammation-Associated Cancers

**DOI:** 10.1371/journal.pone.0006695

**Published:** 2009-08-19

**Authors:** Merav Darash-Yahana, John W. Gillespie, Stephen M. Hewitt, Yun-Yun K. Chen, Shin Maeda, Ilan Stein, Satya P. Singh, Roble B. Bedolla, Amnon Peled, Dean A. Troyer, Eli Pikarsky, Michael Karin, Joshua M. Farber

**Affiliations:** 1 Laboratory of Molecular Immunology, National Institute of Allergy and Infectious Diseases (NIAID), National Institutes of Health (NIH), Bethesda, Maryland, United States of America; 2 SAIC Frederick, National Cancer Institute at Frederick, Frederick, Maryland, United States of America; 3 Tissue Array Research Program, Laboratory of Pathology, Center for Cancer Research (CCR), National Cancer Institute (NCI), National Institutes of Health (NIH), Bethesda, Maryland, United States of America; 4 Institute for Adult Diseases, Asahi Life Foundation, Tokyo, Japan; 5 Hadassah-Hebrew University Medical Center, Jerusalem, Israel; 6 Department of Pathology, University of Texas Health Science Center at San Antonio, San Antonio, Texas, United States of America; 7 University of California San Diego, San Diego, California, United States of America; New York University School of Medicine, United States of America

## Abstract

Clinical observations and mouse models have suggested that inflammation can be pro-tumorigenic. Since chemokines are critical in leukocyte trafficking, we hypothesized that chemokines play essential roles in inflammation-associated cancers. Screening for 37 chemokines in prostate cancer cell lines and xenografts revealed CXCL16, the ligand for the receptor CXCR6, as the most consistently expressed chemokine. Immunohistochemistry and/or immunofluorescence and confocal imaging of 121 human prostate specimens showed that CXCL16 and CXCR6 were co-expressed, both on prostate cancer cells and adjacent T cells. Expression levels of CXCL16 and CXCR6 on cancer cells correlated with poor prognostic features including high-stage and high-grade, and expression also correlated with post-inflammatory changes in the cancer stroma as revealed by loss of alpha-smooth muscle actin. Moreover, CXCL16 enhanced the growth of CXCR6-expressing cancer and primary CD4 T cells. We studied expression of CXCL16 in an additional 461 specimens covering 12 tumor types, and found that CXCL16 was expressed in multiple human cancers associated with inflammation. Our study is the first to describe the expression of CXCL16/CXCR6 on both cancer cells and adjacent T cells in humans, and to demonstrate correlations between CXCL16 and CXCR6 vs. poor both prognostic features and reactive changes in cancer stoma. Taken together, our data suggest that CXCL16 and CXCR6 may mark cancers arising in an inflammatory milieu and mediate pro-tumorigenic effects of inflammation through direct effects on cancer cell growth and by inducing the migration and proliferation of tumor-associated leukocytes.

## Introduction

Recent mouse models have made a functional link between cancer and inflammation [1]–[3], providing evidence for a tumor-promoting activity for leukocytes as originally suggested by Rudolf Virchow [4], [5]. Human prostate cancer has been postulated to arise from precancerous processes associated with inflammation, such as proliferative inflammatory atrophy (PIA) [6], which are lesions found adjacent to prostate cancer that are proposed to lead to prostatic intraepithelial neoplasia (PIN) or cancer [7].

Among the factors important for inflammation are chemokines – chemotactic cytokines that mediate leukocyte recruitment [8]. Diverse roles have been reported for chemokines in tumor biology, including direct effects on cancer cells, such as on transformation, survival, and proliferation, and indirect effects, such as in angiogenesis and in recruiting leukocytes to tumor sites [9]–[11]. In view of the recent evidence for possible pro-tumorigenic activity of leukocytes, we hypothesized that inflammatory chemokines, either produced by the tumor cells or by tumor-associated leukocytes, might, contrary to the standard viewpoint, contribute to malignant progression by enhancing leukocyte recruitment.

In the studies described below, a screen for expression of 37 chemokines in prostate cancer cell lines and xenografts revealed high-level expression of CXCL16, an unusual chemokine that exists in both transmembrane and soluble forms [12], [13], and that can also function as a scavenger receptor for oxidized lipoprotein and bacteria [14]. CXCL16 expression has been associated with a number of human inflammatory diseases including rheumatoid arthritis [15], [16], interstitial lung diseases [17], [18], atherosclerosis [19]–[23], coronary artery disease [24], [25] and liver injury [26]–[30]. The CXCL16 receptor, CXCR6, has been reported to be expressed on Th1 cells [31], on tumor infiltrating lymphocytes [32], and on a variety of leukocytes in inflamed tissue sites [33], [34], and can serve as a co-receptor for HIV-1 [32], [35].

Our study suggests direct roles for CXCL16-CXCR6 on prostate cancer by demonstrating co-localization of CXCL16 and CXCR6 on cancer cells, finding significant positive correlations between expression of CXCL16 and CXCR6 vs. the stage and grade of prostate cancer, and showing that CXCL16 can stimulate the growth of prostate cancer cell lines transfected to express CXCR6.

Our studies on the relation between CXCL16/CXCR6, inflammation, and cancer show that CXCL16 is up-regulated on pre-neoplastic lesions associated with inflammation, that the expression of CXCL16 and CXCR6 can be induced in prostate epithelium by inflammatory cytokines, and that CXCL16/CXCR6 are highest in those prostate cancer cells surrounded by reactive, i.e. post-inflammatory stroma. We also showed that T cells adjacent to cancer cells express CXCL16/CXCR6, that CXCL16 is produced preferentially by CD4^+^CXCR6^+^ T cells, and that CXCL16 can enhance the proliferation of T cells. Finally we demonstrate CXCL16 in multiple human cancers associated with inflammation. Together our data suggest that CXCL16 and CXCR6 may contribute to tumor progression directly through effects on cancer cell growth, and indirectly, by enhancing leukocyte recruitment and proliferation and the interactions between leukocytes and pre-malignant/malignant cells.

## Results

### Malignant prostate cells express CXCL16 and CXCR6

**Figure 1:**
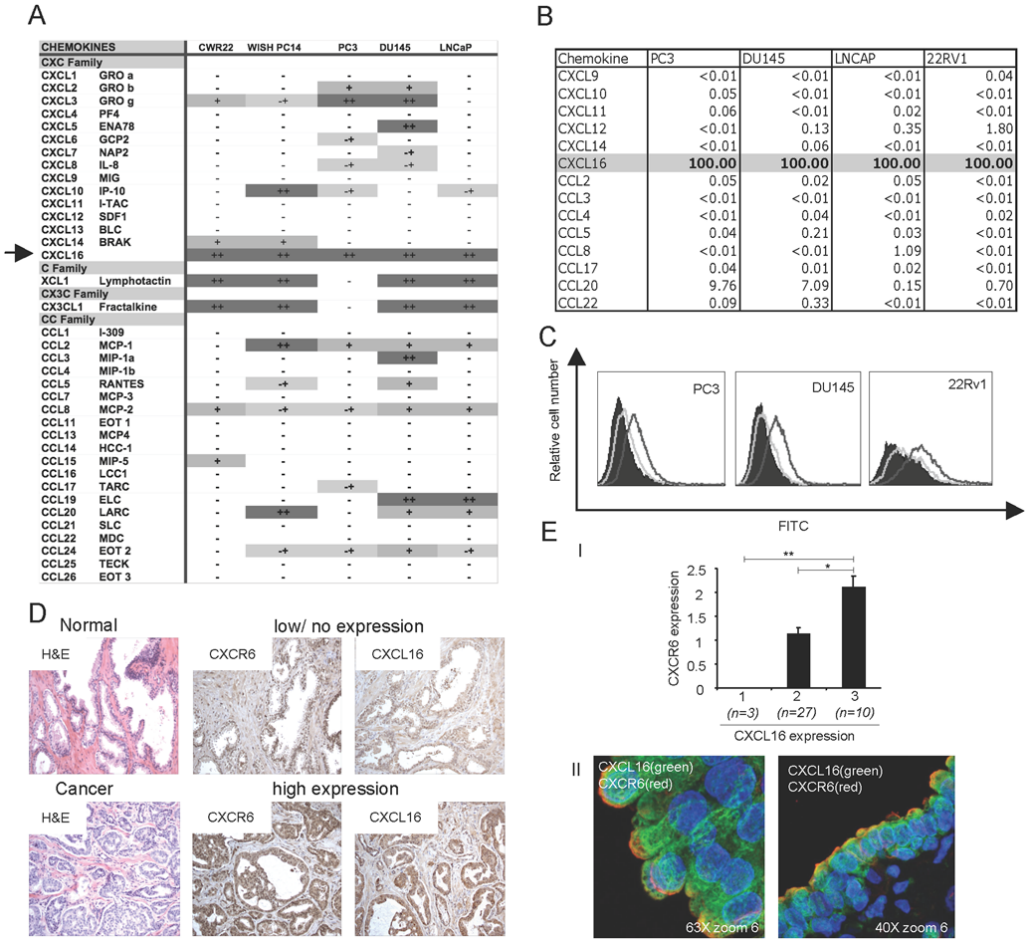
CXCL16 is the most prevalent chemokine expressed in prostate cancer cell lines and xenografts and its expression correlates with levels of CXCR6 on cancer cells. (A) The expression of mRNAs for 37 chemokines was studied by RT-PCR in xenografts (CWR22, WISH PC14) and prostate tumor cell lines (PC3, DU145, LNCaP). Systematic and non-systematic names for chemokines are shown. Amplicons were analyzed by agarose gel electrophoresis and visual inspection after staining with ethidium bromide. Sequences of primers are available on request. The levels of expression were evaluated as follows: no expression (−), low expression (−+), moderate expression (+), and high expression (++). Depending on the chemokine, the analysis was done from one to four times. (B) mRNA expression of chemokines in prostate cancer cell lines. The expression of mRNAs for 14 chemokines was studied by real-time RT-PCR on four prostate tumor cell lines (PC3, DU145, LNCaP, 22Rv1). The value of 2^-ΔCT^ was calculated and then normalized to the highest number for a given cell line, which was given the value of 100, where ΔCT = CT (chemokine)-CT (GAPDH). Numbers are averages of two determinations from one representative experiment of two performed. (C) Flow cytometric analysis of surface expression of CXCL16 on prostate cancer cell lines. PC3, 22Rv1 and DU145 cells were incubated without primary antibody (black shading), with goat anti-human CCL4 as an additional control (light gray line), or with goat anti-human CXCL16 (dark gray line) and then stained with rabbit anti-goat IgG-FITC. Data are from one representative experiment of three performed. (D) Staining for CXCL16 and CXCR6 in prostatectomy specimens using DAB (brown) for detection is shown for: normal prostate and prostate cancer. Panels are representative of over 80 prostate cancers cases examined. Each panel includes an H&E stain on the left. (E) Arrays containing prostate cancer tissue from 40 patients were stained for CXCL16 and CXCR6. (I) Samples were analyzed as described in Materials and Methods, grouped according to scores for CXCL16 on the X-axis (n = samples in each CXCL16 group), and mean scores for CXCR6 expression were calculated. Error bars show SEM. Cross-bars indicate comparisons that are significant. *, p<0.05 and **, p<0.01. (II) Immunofluorescence and confocal imaging of a section showing prostate cancer cells displays anti-CXCL16 (488 tyramide) as green, anti-CXCR6 (594 tyramide) as red, and nuclear staining with Hoechst 33258 as blue. Double staining in yellow shows co-localization. Panels are representative of more than 15 cases of prostate cancer. Original magnifications are noted on these and all subsequent photomicrographs.

In order to investigate the roles of chemokines in prostate cancer, we screened prostate cancer cell lines and xenografts for expression of mRNAs for 37 chemokines, and found that the mRNA for CXCL16 was the most consistently expressed (Figure 1A, B). Cell surface expression of CXCL16 was found on PC3, DU145 and 22Rv1 prostate cell lines (Figure 1C). Immunohistochemical analysis of prostate tissue showed low or no staining for CXCL16 and low staining for its receptor, CXCR6 in normal human prostate epithelium, but prominent expression in the cancer (Figure 1D). In addition, a correlation between the expression levels of CXCL16 and CXCR6 was found by analyzing an array of forty cases of prostate cancer, and CXCL16 and CXCR6 can be shown to be co-expressed on individual cancer cells by immunofluorescent confocal microscopy (Figure 1E).

### Expression of CXCL16 and CXCR6 correlates with cancer stage and grade

**Figure 2:**
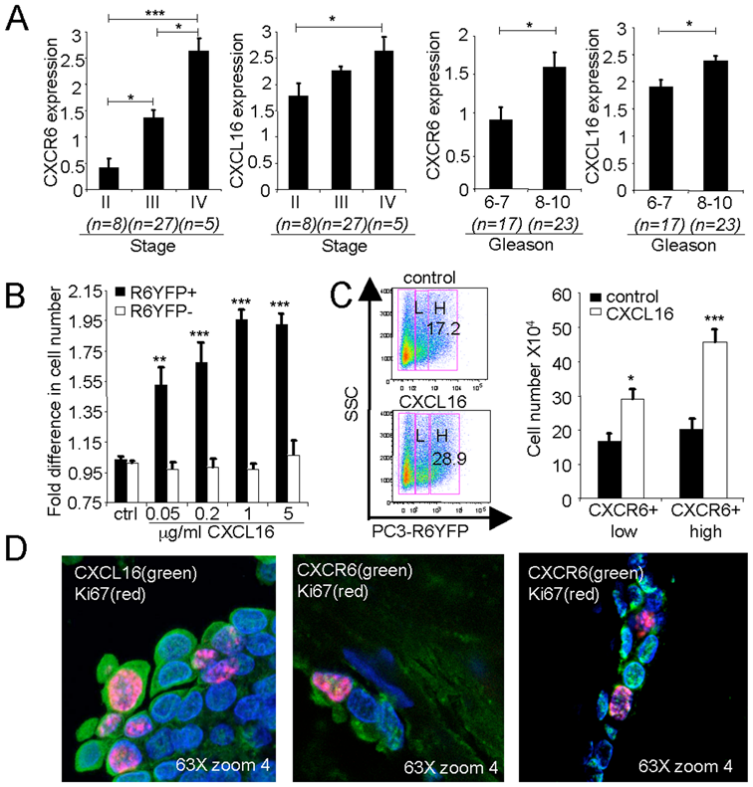
Roles for CXCL16 and CXCR6 in prostate cancer stage, grade and cell growth. (A) Arrays containing prostate cancer tissue from 40 patients were stained for CXCL16 and CXCR6. Samples were scored for CXCL16 and CXCR6 expression as described in Materials and Methods. Samples were grouped according to stage or Gleason score (X-axes) as determined by the supplier, and mean scores for CXCL16 and CXCR6 expression were calculated. The number of samples (n) in each stage or Gleason score group is shown. Error bars show SEM. Cross-bars indicate comparisons that are significant. *, p<0.05 and ***, p<0.001. (B) PC3 cells were transfected with the CXCR6-YFP plasmid, cultured for 48 hours without chemokine (control, ctrl) or with 0.05–5 µg/ml CXCL16 and numbers of CXCR6-YFP^+^ (R6YFP^+^) vs. CXCR6YFP^−^ (R6YFP^−^) cells were counted as described in Materials and Methods. Values were normalized to the control, untreated sample to yield the fold difference in cell number. Bars show means±SEM combined from three experiments. **, p<0.01 and ***, p<0.001 vs. control values. (C) Following transfection with the CXCR6-YFP plasmid, PC3 cells were cultured as in (B), without or with 5 µg/ml CXCL16. Left panel, gating is shown for separating untreated and CXCL16-treated cells into “low” (L) or “high” (H) for CXCR6-YFP. Percentages of cells in the high gates are shown. One representative experiment is shown out of three performed. Right panel, CXCR6-YFP^+^low (CXCR6+low) and CXCR6-YFP^+^high (CXCR6+high) cells from CXCL16-treated and control cultures were counted after 48 hours as in (B). Data were combined from three experiments. *, p<0.05 and ***, p<0.001 vs. untreated cells. (D) Confocal imaging displays anti-CXCL16 or anti-CXCR6 (488 tyramide) as green, anti-Ki67 (594 tyramide) as red, and nuclear staining with Hoechst 33258 as blue. Panel is representative of more than 15 cases.

The stage (I-IV) and grade (Gleason score) of prostate cancer are determined at diagnosis, and were reported by the supplier for the cases used in the arrays. Higher stage corresponds to greater anatomic extension of the cancer, and Gleason score describes histological characteristics that correlate with clinical outcome. Gleason scores of 2–4 are low-grade, scores 5–7 are moderate-grade, and scores 8–10 are high-grade prostate cancer. Analysis of the 40-case array for levels of staining for CXCL16 and CXCR6 revealed that their expression by the cancer cells correlated with both high-stage and high-grade prostate cancer (Figure 2A).

### A role for CXCL16/CXCR6 in the proliferation of prostate cancer cells

The co-expression of CXCL16 and CXCR6 on prostate cancer cells, and their correlation with cancer stage and grade, suggested that the ligand and receptor might enhance proliferation through an autocrine pathway. To investigate this possibility, we transfected PC3 cells with a CXCR6-YFP plasmid that encodes a CXCR6 C-terminal fusion protein with an enhanced yellow-green florescent protein (YFP), and incubated the cells with CXCL16. As shown in Figure 2B, the number of CXCR6-YFP^+^ cells increased, vs. control CXCR6-YFP^−^ cells, as a function of the concentration of CXCL16. We also analyzed the relationship between expression level of CXCR6-YFP and the response to CXCL16. We found that while cells expressing the highest levels of CXCR6-YFP were those most responsive to CXCL16, CXCL16 increased the proliferation even of those cells that were CXCR6-YFP^low^ (Figure 2C). Consistent with these in vitro data, cancer cells expressing Ki67, a cellular marker for proliferation, also expressed both CXCL16 and CXCR6 (Figure 2D).

### Expression of CXCL16/CXCR6 is associated with inflammation

**Figure 3:**
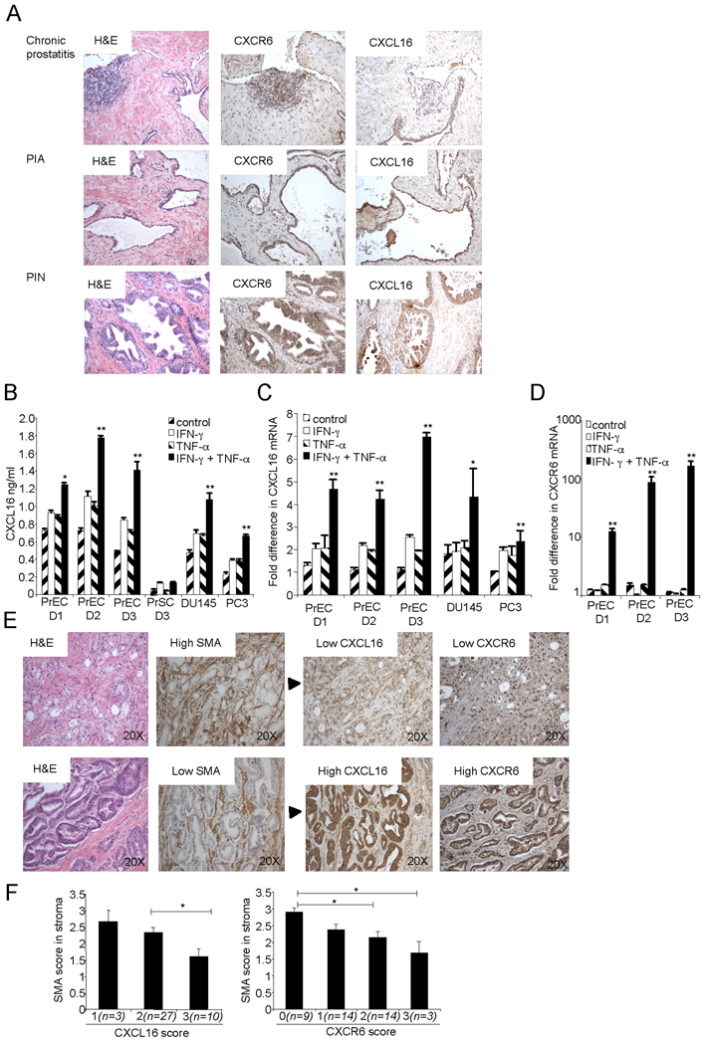
Inflammatory stroma and cytokines induce CXCL16 and CXCR6 on normal and malignant prostate cells. (A) Staining for CXCL16 and CXCR6 in prostatectomy specimens using DAB (brown) for detection is shown for: chronic prostatitis, PIA and PIN. Panels are representative of over 80 prostate cancer cases examined. Each panel includes an H&E stain on the left. (B) CXCL16 secreted by primary normal epithelial cells (PrEC) and stromal cells (PrSC), and the DU145 and PC3 cell lines was measured 48 hours after no treatment (control), or treatment with 5 ng/ml IFN-γ and 0.5 µg/ml TNF-α alone and in combination. Bars show means±SEM from duplicate wells, of one representative experiment of three performed for each of three donors (D1–D3). *, p<0.05 and **, p<0.01 vs. untreated cells. (C) CXCL16 mRNA expression in PrEC from three donors and two prostate tumor cell lines (DU145 and PC3) treated with 5 ng/ml IFN-γ and 0.5 µg/ml TNF-α alone and in combination were analyzed using real-time RT-PCR. Values were normalized by setting the control sample with lowest expression equal to 1. Each bar represents the mean±SEM obtained from duplicate wells, from one representative experiment of three performed. **, p<0.01 vs. untreated cells. (D) CXCR6 mRNA expression in PrEC from three donors treated with 5 ng/ml IFN-γ and 0.5 µg/ml TNF-α alone and in combination were analyzed using real-time RT-PCR. Values were normalized by setting the sample with lowest expression equal to 1. **, p<0.01 vs. untreated cells. (E) Each row contains serial sections from the same regions of prostatectomy specimens stained using DAB (brown) for αSMA, CXCL16, and CXCR6 including an H&E stain on the left. Panels are representative of over 120 prostate specimens. (F) Separate slides of arrays containing prostate tissue from 40 patients were stained for αSMA, CXCL16 and CXCR6 and scored as described in Material and Methods for αSMA in stroma and CXCL16 and CXCR6 in cancer cells. Samples were grouped according to scores for CXCL16 or CXCR6 (n = samples in each group), and mean scores were calculated for αSMA in stroma. Data are displayed and analyzed as in Fig. 1E.I. *, p<0.05.

Immunohistochemical analysis of multiple specimens from radical prostatectomies showed prominent expression of CXCL16 and CXCR6 in chronic prostatitis and in atrophy with associated chronic prostatitis (PIA), as well as in prostatic intraepithelial neoplasia (PIN) and cancer, suggesting that expression of CXCL16 and CXCR6 on prostate epithelial cells is related to inflammation in addition to any association with malignancy per se (Figure 3A). We investigated this relationship in vitro by treating cultures of epithelial (PrEC) and stromal cells (PrSC) from normal prostates with inflammatory cytokines. TNF-α and IFN-γ, particularly in combination, induced CXCL16 mRNA and CXCL16 secretion by PrEC (but not stromal cells) (Figure 3B, C) – and led to dramatic up-regulation in PrEC of the mRNA for CXCR6 (Figure 3D).

In addition to the association of CXCL16/CXCR6 with inflammatory infiltrates in prostate, our initial impression from analyzing more than 80 cases of prostate cancer was that expression of CXCL16 was highest in cancer cells surrounded by reactive stroma. Stroma is described as “reactive” if it shows increases in myofibroblasts, fibroblast to smooth muscle ratio and local vascular density [36]. This occurs following inflammation, and identifies cancers, now relatively poor in inflammatory cells, that have arisen in an inflammatory micro-environment. We confirmed our initial impression by staining serial sections for CXCL16, CXCR6 and alpha smooth muscle actin (α-SMA), a marker for smooth muscle cells, which are lost from reactive stroma. We found an association between loss of smooth muscle cells and high expression of CXCL16 and CXCR6 (Figure 3E), which was confirmed by scoring samples in the 40-case array for α-SMA, CXCL16, and CXCR6 (Figure 3F). These data supported the association between expression of CXCL16 and CXCR6 and evidence of prior inflammation in the tumor micro-environment. Together, the data in Figure 3 suggest a strong correlation between expression of CXCL16/CXCR6 and inflammation at all stages in the evolution of prostate cancer.

### A role for CXCL16/CXCR6 in interactions between cancer cells and T cells

**Figure 4:**
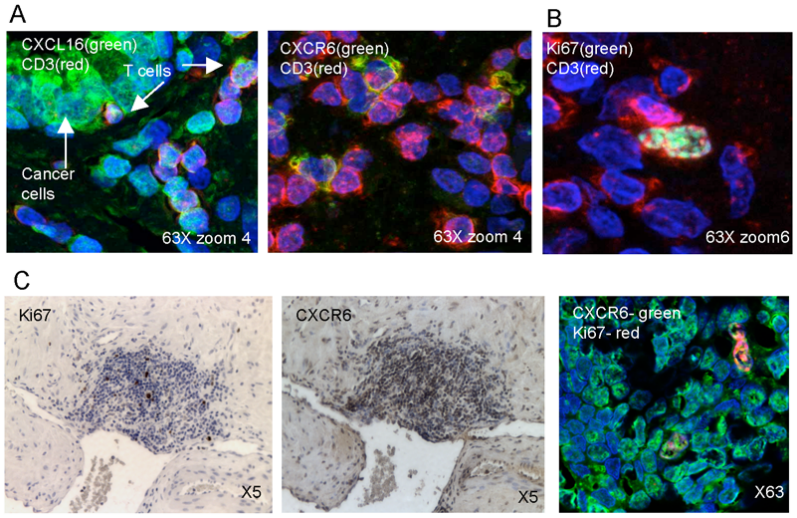
CXCL16 and CXCR6 are expressed by T cells in prostate tissue. (A, B) Confocal imaging shows anti-CXCL16 (A, left panel) or anti-CXCR6 (A, right panel) or anti-Ki67 (B) as green (488 tyramide), anti-CD3 (594 tyramide) as red, and nuclear staining with Hoechst 33258 as blue. Double staining in yellow shows co-localization. In (A), arrows point to cancer cells and T cells. Panels are representative of more than 15 cases for each double-stain. (C) Staining for Ki67 and CXCR6 in a region of prostatitis using DAB (brown, two left panels) or immunofluorescence (right panel) for detection. Confocal imaging shows anti-CXCR6 as green (488 tyramide) and anti-Ki67 as red (594 tyramide), and nuclear staining with Hoechst 33258 as blue. Panel is representative of more than 15 cases.

The possible direct effects of CXCL16/CXCR6 on prostate cancer cells notwithstanding, because CXCR6 is best described as a T cell chemokine receptor, we investigated activities for CXCL16/CXCR6 on prostate-cancer associated T cells that might contribute indirectly to the formation and growth of the cancer. Immunofluorescent staining revealed that both CXCL16 and CXCR6 are expressed on CD3^+^ cells adjacent to cancer cells (Figure 4A). Of interest, we found that some CD3^+^ cells also stained for Ki67 (Figure 4B), demonstrating T cell proliferation at the tumor site. Moreover, double staining for CXCR6 and Ki67 revealed inflammatory cells that were positive for both (Figure 4C).

**Figure 5:**
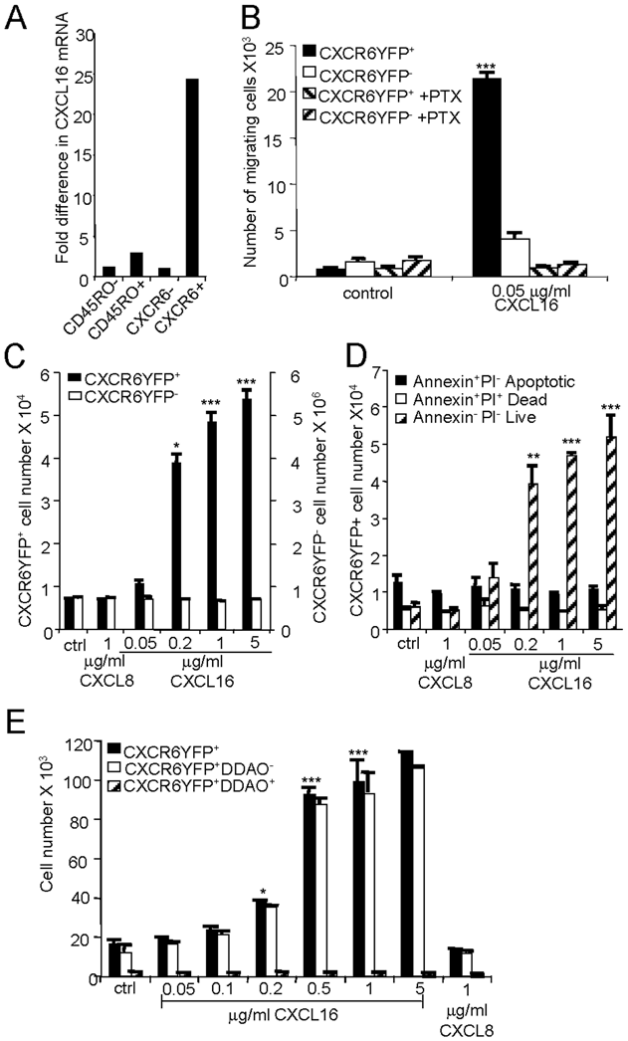
CXCL16 can mediate the migration and proliferation of T cells expressing CXCR6. (A) CD4^+^ T cells were sorted into naïve (CD45RO-), total effector/memory (CD45RO+), CD45RO^+^CXCR6^−^ (CXCR6-) and CD45RO^+^CXCR6^+^ (CXCR6+) subsets and stimulated with PMA and ionomycin for 4 hours before measuring levels of CXCL16 mRNA by real-time RT-PCR. Values were normalized by setting the sample with lowest expression equal to 1. The data are means from duplicate samples from one representative experiment out of three performed. (B) Jurkat E6.1 T cells transfected with the CXCR6-YFP plasmid were analyzed for migration to CXCL16. Some samples were pre-incubated for two hours with 400 ng/ml pertussis toxin as indicated. Numbers of YFP+ and YFP- migrating cells were analyzed by flow cytometry. Each bar represents the mean±SEM obtained from triplicate wells, from one representative experiment of three performed. ***, p<0.001 vs. the corresponding control value. (C) Following transfection with the CXCR6-YFP plasmid, Jurkat E6.1 T cells were cultured for 48 hours without chemokine (control, ctrl) or with 1 µg/ml CXCL8 as an additional control, or with 0.05–5 µg/ml CXCL16 before counting numbers of CXCR6-YFP^+^ vs. CXCR6-YFP^−^ cells. Bars show means±SEM combined from three different experiments. *, p<0.05 and ***, p<0.001. (D) Transfected cells were treated as in (C) and stained with Annexin V and propidium iodide (PI) before counting numbers of CXCR6-YFP^+^ cells within the subgroups as noted. Bars show means±SEM combined from three experiments. **, p<0.01 and ***, p<0.001 vs. control untreated cells. (E) Following transfections with CXCR6-YFP plasmid, Jurkat E6.1 T cells were loaded with 2 µM Far Red DDAO dye and cultured without chemokine (control, ctrl) or with 0.05–5 µg/ml CXCL16 or 1 µg/ml CXCL8. Numbers of CXCR6YFP^+^ vs. CXCR6YFP^+^DDAO^−^ (proliferated) and CXCR6YFP^+^DDAO^+^ (non-proliferated) cells were determined after 48 hours using counting beads and flow cytometry. Bars show means±SEM from triplicate wells, from one representative experiment of three performed. ***, p<0.001 and *, p<0.05 vs. control untreated cells.

We considered whether CXCL16 and CXCR6 might function in an autocrine fashion to enhance T cell proliferation analogous to effects on cancer cells. To determine whether or not CXCL16 could be produced by CXCR6^+^ cells, which are found only in the effector/memory subset, we sorted populations of peripheral blood CD4^+^ T cells based on the expression of memory and naïve markers and CXCR6. After activation *ex vivo*, we found that the mRNA for CXCL16 was induced preferentially within the effector/memory subset and, within that subset, in the cells expressing CXCR6 (Figure 5A). We next tested the potential activities of CXCR6 on T cells by transfecting the Jurkat E6.1 T cell line with DNA encoding CXCR6-YFP or GFP. CXCR6-YFP^+^, but not CXCR6-YFP^−^ cells, migrated to CXCL16 in a G_i/o_-protein dependent fashion (Figure 5B), and showed a progressive increase in cell numbers with increasing doses of CXCL16, but not to a control chemokine (Figure 5C). CXCL16 had no effect on cells transfected to express GFP as an additional control (data not shown). CXCL16/CXCR6 enhanced proliferation per se, since we saw no effects on cell death (Figure 5D) and found an increase specifically within the population of cells that were dividing ( Figure 5E).

**Figure 6:**
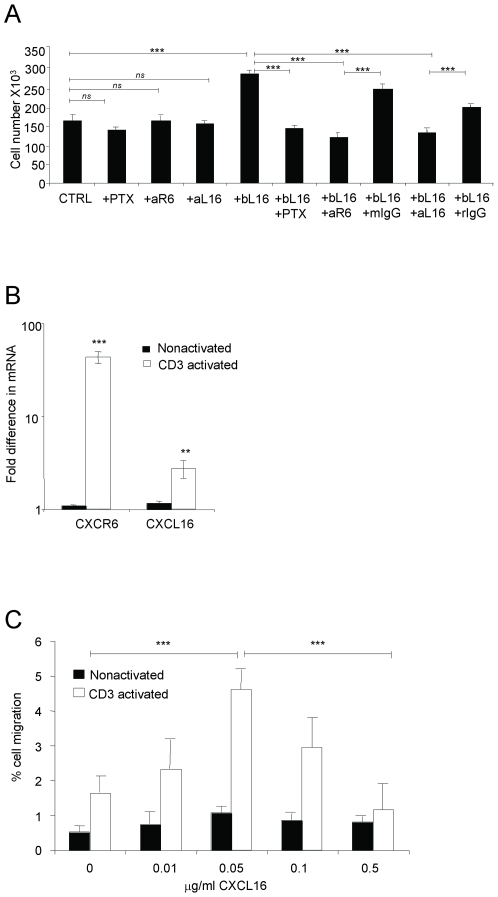
CXCL16 can mediate the proliferation and migration of CD3-activated primary CD4 T cells. (A) CD4^+^ T cells purified from elutriated lymphocytes from healthy donors were stimulated for 3 days with plate-bound anti-CD3 (OKT3, 10 µg/ml) with or without 5 µg/ml plate-bound CXCL16 (bL16). Treatments of anti-CD3-activated cells with PTX, anti-CXCR6 (aR6) and anti-CXCL16 (aL16) without bL16 were done as controls. Mouse IgG (mIgG) and rat IgG (rIgG) were used as controls for anti-CXCR6 antibody and anti-CXCL16 antibody, respectively. Bars show means±SEM from one representative experiment out of five, using five donors. Anti-CXCL16 was used in a total of four, and anti-CXCR6 in two experiments. ns, not significant and ***, p<0.001. (B) CXCR6 and CXCL16 mRNAs were measured by real-time RT-PCR in CD3-activated and non-activated CD4^+^ T cells after 3 days. Values were normalized by setting the non-activated sample with lowest expression equal to 1. Bars show means±SEM combined from duplicate wells from seven different donors. **, p<0.01 and ***, p<0.001 vs. non-activated cells. (C) Anti-CD3-activated or nonactivated CD4^+^ T cells were analyzed for migration to CXCL16, expressed as percentages of input cells migrating. Each bar represents the mean±SEM obtained from triplicate wells from a total of three experiments performed. ***, p<0.001 on cross bars are indicated for comparisons between activated cells - 0 vs. 50 ng/ml CXCL16 and 50 ng/ml vs. 500 ng/ml CXCL16.

Moreover, when we examined the effect of plate-bound CXCL16 on the proliferation of CD3-activated primary CD4 T cells, we found a significant stimulatory effect on proliferation that was inhibited by treating with pertussis toxin or antibodies against CXCR6 or CXCL16 (Figure 6A). It is notable, given that CXCL16 is synthesized as a transmembrane chemokine, that although plate-bound CXCL16 was stimulatory, soluble CXCL16 had no effect (data not shown).

Despite the data for effects of CXCL16 on activated CD4^+^ T cells, we could not detect staining for CXCR6 using flow cytometry on a large or increased percentage of these cells after activation. However, in support of functional CXCR6 on these cells, we found a significant increase in mRNA expression of CXCR6 and (less so) of CXCL16 in CD3-activated cells (Figure 6B), and an increase in the migration of the CD3-activated cells to CXCL16 (Figure 6C). The dose response in Figure 6C has the bell-shaped pattern typical for chemotaxis.

### CXCL16 and/or CXCR6 are broadly expressed in inflammation-associated cancers

**Figure 7:**
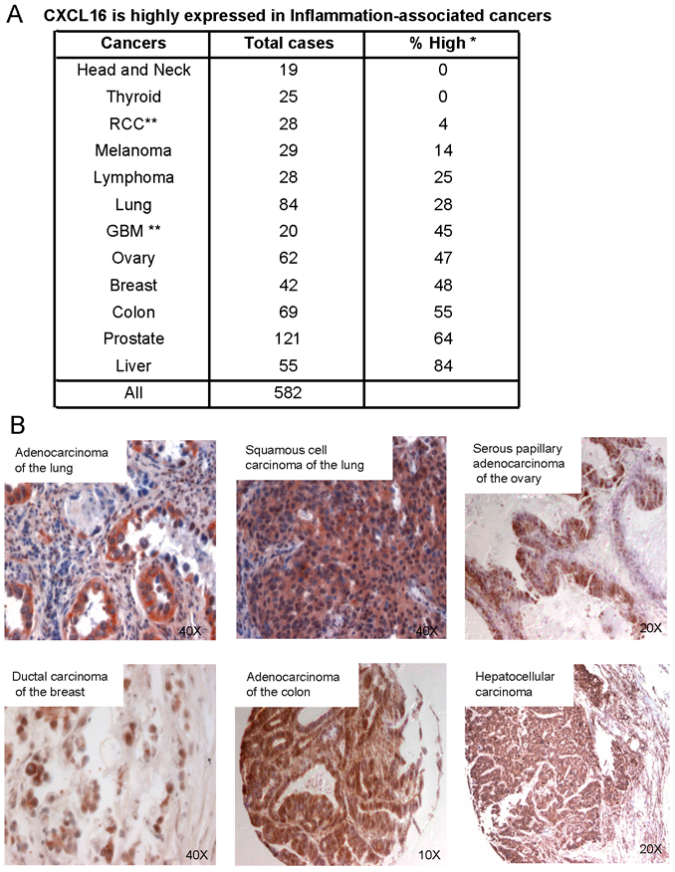
CXCL16 is highly expressed in multiple inflammation-associated cancers. (A) * The intensity of CXCL16 staining was scored as described in Materials and Methods. The % of cases staining high is shown for each cancer type. ** RCC, renal cell carcinoma; GBM, glioblastoma multiforme. (B) CXCL16 expression in arrays of multiple human cancers by immunohistochemistry using AEC (red) or DAB (brown) for detection. Panels are representative of the numbers of specimens of the various cancers as listed in (A).

In order to investigate the possibility of a more general association between CXCL16 and inflammation-associated cancer, we studied the expression of CXCL16 in 582 cases of cancer covering 12 tumor types (Figure 7). We found a bias for high CXCL16 expression in cancers associated with inflammation: ovary, breast, prostate, colon and liver cancers.

## Discussion

Cancer arises from genetic mutations and epigenetic events in the proliferating cells and changes in the tumor's microenvironment that includes capillaries, smooth muscle cells, fibroblasts and inflammatory cells [4]. In 1863, the German physician Rudolf Virchow noted leukocytes in neoplastic tissues and hypothesized that cancer begins at sites of chronic inflammation [37]. Nonetheless, cancer immunologists have generally viewed leukocytes as agents with the potential to limit or eliminate cancers [38]. Similarly, in their roles as mediators of leukocyte recruitment, chemokines have been studied as stimulators of anti-cancer immunity and inflammation [11], [39], [40].

Chronic inflammatory conditions have, however, been shown to predispose to multiple cancer types and recurrent or chronic inflammation has been implicated in a variety of human cancers [5]. Recent mouse models have made a functional link between cancer and inflammation, showing that deletion of immune cells or immunoregulatory genes reduce cancer progression [1]–[3]. Together, these data raise the possibility of an additional role for pro-inflammatory chemokines in cancer, namely as tumor promoters.

We investigated prostate cancer, which is described as arising in the context of inflammation. Our preliminary screen for chemokine expression suggested a role for CXCL16, and we found that both CXCL16 and CXCR6 were co-expressed on cancer cells, that their levels of expression correlated with each other, and that these levels also correlated with high-stage and high-grade prostate cancer. In addition, CXCL16 enhanced proliferation of prostate cancer cell lines expressing CXCR6. Our data suggest possible direct effects of CXCL16/CXCR6 on cancer cell growth and thereby on tumor development.

In a second aspect of our study, we investigated potential roles for CXCL16/CXCR6 within the context of the postulated contribution of inflammation to pre-malignant and malignant disease in the prostate. We found high expression for CXCL16 in chronic prostatitis, pre-neoplastic lesions, and primary prostate cancer surrounded by reactive, i.e. post-inflammatory stroma. Moreover, TNF-α and IFN-γ induced the expression of CXCL16 and CXCR6 on primary prostate epithelial cells. In addition, we found significant correlations between the presence of reactive stroma and high-level expression of CXCL16 and CXCR6 in cancer cells. These results suggested that up-regulation of CXCL16/CXCR6 in prostate epithelium is strongly associated with both current and previous inflammation in prostate stroma.

In a third part of our study, we investigated potential roles for CXCL16 and CXCR6 on tumor-associated lymphocytes. We found that CXCL16 and CXCR6 are expressed on T cells adjacent to prostate cancer cells. We demonstrated that CXCL16 can be induced preferentially in CXCR6^+^CD4^+^ primary T cells, and that Jurkat T cells transfected to express CXCR6 as well as activated, primary CD4^+^ T cells proliferated in the presence of CXCL16. These data suggest the potential for both autocrine and paracrine effects of CXCL16 on infiltrating T cells as well as on pre-malignant and/or malignant prostate cells.

Finally, to investigate a more general association between CXCL16 and inflammation-associated cancers, we studied the expression of this chemokine in specimens from multiple types of human cancers. We found high expression levels of CXCL16 in cancers of the ovary, breast, prostate, colon and liver – all cancers described as arising in the context of inflammation.

CXCL16 has been previously described on human gliomas, colon and breast cancer, and CXCR6 was reported in nasopharyngeal cancers and melanoma [41]–[46]. Several additional papers have recently appeared investigating CXCL16 and/or CXCR6 in cancer [45]–[50]. Several of these reports focused on CXCR6 in prostate cancer, and like ours showed high expression of CXCR6 in prostate cancer cells [47]–[49]. One paper, also like ours, demonstrated a positive correlation between CXCR6 levels and Gleason scores, reported CXCL16 expression in prostate cancers, and showed increased secretion of CXCL16 from prostate cell lines after treating with TNF-α [47]. A second paper showed effects on invasion of prostate lines to soluble CXCL16 [48]. A third paper demonstrated effects of CXCR6 on the growth of prostate cancer cell lines in mice [49]. Overall, our findings suggesting pro-tumorigenic roles for CXCL16 and CXCR6 in prostate cancer are consistent with these published reports. These manuscripts did not, however, investigate expression of, or roles for CXCL16/CXCR6 in cancer-associated inflammation or leukocytes, or in the pre-malignant stages of prostate cancer. Of additional related interest, a recent study reported induction of CXCL16 in bone stroma conditioned by prostate cancer cells, and elevated serum levels of CXCL16 in patients with metastatic prostate cancer [50].

It is notable that in experiments using colon carcinoma cell lines, membrane-bound CXCL16 inhibited cell proliferation [45] and in a study of CXCL16 in colon cancer, no association was found between CXCL16 expression and cancer stage - but instead a positive correlation was reported between CXCL16 expression and patient survival [42]. This latter observation contrasts with our findings on the positive correlations between expression of CXCL16 and CXCR6 and high stage and grade for prostate cancer, and may reflect variable roles for inflammation in cancer depending on tumor type and stage of tumor development.

To date, multiple chemokines have been described having roles in tumor growth and metastasis [51]. Our study is the first to describe expression of the chemokine CXCL16 and its receptor CXCR6 on both cancer cells and adjacent T cells in human cancer and demonstrate correlations between expression of CXCL16 and CXCR6 and tumor progression. By demonstrating a correlation between this chemokine-receptor pair and reactive, post-inflammatory stroma, our study is also the first to make a connection between expression of a chemokine and its receptor and changes in the microenvironment of human cancer.

**Figure 8:**
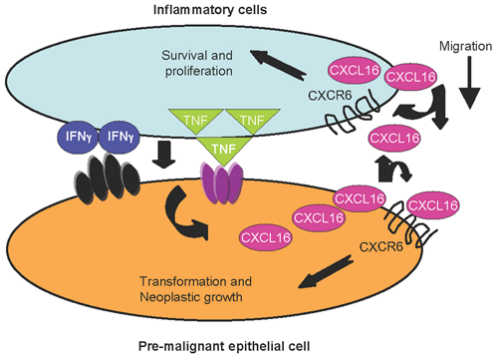
CXCL16 and CXCR6 establish positive feedback loops contributing to the progression of inflammation-associated cancers. Secretion of CXCL16 from pre-cancer or cancer cells leads to CXCR6-mediated recruitment of leukocytes. Cytokines such as TNF-α and IFN-γ produced by these cells in turn induce expression of CXCL16 and CXCR6, creating a positive feedback loop that enhances tumorigenesis – directly, through effects on the growth of pre-cancer and cancer cells, and indirectly, by increasing the migration and proliferation of the inflammatory cells, together leading to the increased growth of inflammation-associated cancers.

Taken together, our data suggest that CXCL16 and CXCR6, expressed by both inflammatory cells and cancer cells in human cancers, may be components of inter-related paracrine and autocrine positive feedback loops involving these cells, pleiotropic cytokines and chemokines. CXCL16 could enhance tumor growth directly through CXCR6-mediated effects on the proliferation/survival of cancer cells and indirectly through enhanced recruitment and proliferation/survival of cytokine-producing T cells (Figure 8). Our data correlating CXCL16 and CXCR6 with stage and grade of prostate cancer provide additional evidence that, in the prostate, inflammation is tumorigenic, and should stimulate the increasing interest in a possible tumor-promoting role specifically for CD4^+^ T cells [52]. Although we have focused on prostate cancer, our findings that multiple inflammation-associated human cancers express high levels of CXCL16 and CXCR6 suggest that this chemokine/receptor pair may serve as a marker of current or prior inflammation within cancers, thereby providing a tool that can be broadly applied to studying the relationships between inflammation and the behaviors of various malignancies.

## Methods

### Cells and Tissues

PrEC and PrSC were purchased from Cambrex Bio Science, Walkersville, MD, and the PC3, DU145, 22Rv1, LNCaP and Jurkat cell lines were purchased from American Type Culture Collection, Manassas, VA. Cells were grown as instructed by the suppliers.

These samples were obtained from the clinical frozen tissue archives of the Department of Pathology at The University of Virginia: Breast (12 cases), prostate (13 cases), colon (9 cases), ovary (12 cases) and lung (14 cases). There were no specific inclusions or exclusions. The samples were taken from the archives with all identifiers removed after review and approval by the Institutional Review Board for Health Sciences Research (IRB-HSR) at The University of Virginia, which granted a waiver for obtaining informed consent. Additional prostate tissue samples (13 cases) were obtained from the National Cancer Institute and the University of Texas, San Antonio (Tissue Bank #HSC20050234H IRB Protocol #45-8000-234). There were no specific inclusions or exclusions. The samples were taken from the archives with all identifiers removed after review and approval by the Tissue Bank and Data Base for Urologic Diseases of the National Cancer Institute, and the University of Texas, San Antonio IRB, which granted a waiver for obtaining informed consent.

Elutriated lymphocytes were obtained from healthy donors by the Department of Transfusion Medicine, under a protocol (99-CC-0168) approved by the IRB of the Clinical Center, NIH. All blood components collected under this protocol are used for research purposes, to which donors provide written consent. Some tissue arrays were from the Tissue Array Research Program (TARP), Center for Cancer Research, National Cancer Institute and some were purchased from Imgenex, San Diego, CA. The TARP is a biobank. For the TARP arrays, tissues were obtained retrospectively and anonymously from surgical pathology specimens. Patients consented to donation, and samples were transferred to TARP without identifiers. This use was considered exempt from IRB review by the NIH Office of Human Subjects Research. The numbers of samples (as presented also in Figure 7) and the sources for each tumor type were: head and neck cancer - 19 cases, TARP array; thyroid cancer - 25 cases, TARP array; renal cell carcinoma (RCC)- 28 cases, TARP array; melanoma- 29 cases, TARP array; lymphoma- 28 cases, TARP array; lung cancer - 84 cases total - 70 cases from TARP array and 14 cases from University of Virginia; glioblastoma (GBM) – 20 cases, TARP array; ovarian cancer – 62 cases total - 50 from TARP array and 12 from University of Virginia; breast cancer - 42 cases total- 30 cases from TARP array and 12 cases from University of Virginia; colon cancer - 69 cases total- 60 cases from TARP array and 9 cases from University of Virginia; prostate cancer -121 cases total- 75 from TARP array, 13 from University of Texas, 13 from University of Virginia, and 20 from Imgenex commercial array; liver cancer - 55 cases, Imgenex commercial array.

### Antibodies and Cytokines

All antibodies were against human antigens. For immunohistochemistry the following antibodies were used: goat anti-CXCL16 (biotinylated and non-biotinylated, R&D Systems, Minneapolis, MN), rabbit anti-CXCR6 (GeneTex, San Antonio, TX), rabbit anti-CD3, mouse anti-CD3 (F7.2.38), mouse anti-alpha smooth muscle actin (αSMA) (1A4), and mouse anti-Ki-67 (MIB-1) (DakoCytomation, Carpinteria, CA). For each of these antibodies, the corresponding control antibodies, goat, rabbit or mouse IgG were used (R&D Systems). For FACS sorting and/or analysis the following antibodies were used: mouse anti-CXCR6-PE (R&D Systems), mouse anti-CD4-APCCy7 and mouse anti-CD45RO-PECy5 (BD Pharmingen, San Diego, CA). For CD4 proliferation experiments the following antibodies were used: mouse anti-CXCR6 and rat anti-CXCL16 and the corresponding control mouse or rat IgG's (R&D Systems). Cytokines were purchased from R&D Systems. The hybridoma producing anti-CD3 (clone OKT3) was obtained from Ortho Biotech (Raritan, NJ) and antibody was purified from ascites by the Research Technologies Branch, NIAID.

### Real Time RT-PCR

Target cDNA sequences were amplified using “inventoried” primer-probe sets that spanned exon boundaries and using fluorescence-based real-time detection instrumentation (ABI PRISM 7700) from Applied Biosystems, Foster City, CA. Relative gene expression was calculated using the delta-CT method [53]. A control mRNA, for either glyceraldehyde 3-phosphate dehydrogenase (GAPDH) or β2-microglobulin, was analyzed in each run. Primers/probes produced exponential amplification curves.

### Immunohistochemistry

Tissue samples were either snap-frozen or formalin/ethanol-fixed and paraffin-embedded. Frozen tissue was fixed in formalin prior to staining. Paraffin embedded sections were treated for antigen retrieval (Target Retrieval Solution, DakoCytomation). If necessary, endogenous peroxidase was inactivated using Peroxidase Blocking Reagent, followed by blocking endogenous biotin using the Avidin-Biotin Blocking kit (both from DakoCytomation). In some cases, biotinylated rabbit anti-goat IgG secondary antibody was used, and detection was done using a standard avidin–biotin horseradish peroxidase procedure according to the manufacturer's instructions (Zymed Laboratories, San Francisco, CA). 3-amino-9-ethylcarbazole (AEC) or 3,3′-diaminobenzedine (DAB) (DakoCytomation) was used for color development, and sections were counterstained with hematoxylin. For negative controls, sections were stained using rabbit IgG or biotinylated goat IgG (R&D Systems) as appropriate. Staining for CXCL16, CXCR6 and αSMA were scored separately by a single pathologist (J.W.G) blind to clinical data and to results with other stains. For CXCL16 and CXCR6, expression was scored from 0–3, with 0 being negative and 3 the highest level of staining intensity. For αSMA, the percentage of stromal cells staining positive for αSMA was scored with 0 = 0–25%, 1 = 25–50%, 2 = 50–75%, and 3 = 75–100%. For analyzing CXCL16 expression in multiple tumor types (Figure 7), the intensity of CXCL16 staining was scored as “high” (scores 2, 3) or “low” (scores 0, 1) by visual inspection by a single pathologist (J.W.G) and confirmed by a second (S.M.H).

### Immunofluorescence

Immunofluorescence was done using Alexa Flour 488 tyramide and Alexa Flour 594 tyramide (Invitrogen, Eugene, OR). For double staining, horse radish peroxidase used for visualizing the first antibody was deactivated by treatment with 3% H_2_O_2_. The second primary antibody was detected using peroxidase-conjugated F(ab')_2_ donkey anti-rabbit or anti-mouse IgG(H+L) (Jackson IR Laboratories, West Grove, PA) followed by Alexa Flour 594 tyramide. Nuclei were identified using Hoechst 33258 (Sigma-Aldrich, St. Louis, MO). Images were collected on a Leica SP2/AOBS confocal microscope using a 63×or 40×oil immersion objective NA 1.4, and processed using Leica TCS-SP software (version 2.1537, Leica Microsystems, Exton, PA).

### Enzyme-linked Immunosorbent Assay (ELISA)

CXCL16 was measured using a DuoSet ELISA Development kit (R&D Systems), according to the manufacturer's instructions.

### Sorting of Lymphocyte Subsets and Stimulation

CD4^+^ T cells were purified from elutriated lymphocytes by negative selection with the RosetteSep reagents (StemCell Technologies, Vancouver, BC). Subsets were sorted after staining with fluorophore-conjugated anti-CD4, anti-CD45RO, and anti-CXCR6. Cell sorting was done using a FACS Aria (BD Biosciences), and the purity of sorted populations was 95–99%. After sorting, cells were stimulated with 20 ng/ml PMA, and 1 µM ionomycin for 4 hours at 37°C before total cellular RNA was isolated.

### Proliferation of primary CD4^+^ T cells with CXCL16

CD4^+^ T cells purified from elutriated lymphocytes from healthy donors were cultured in RPMI 1640 supplemented with 10% fetal bovine serum, 2 mM L-glutamine, penicillin and streptomycin. Cells were stimulated for 3 days with plate-bound OKT3 (10 µg/ml) with or without plate bound CXCL16 (PeproTech, Rocky Hill, NJ) (5 µg/ml). Samples were analyzed using an LSRII flow cytometer (BD Biosciences) after adding counting beads (SPHERO^TM^ AccuCount Fluorescent Particles, Spherotech Inc, Libertyville, IL) together with 5 µl of Dead Cell Discriminator (CALTAG Laboratories, Burlingame, CA). Numbers of proliferating cells were determined using FlowJo software (Tree Star, Ashland, OR).

### Migration of cells to CXCL16

Cells were washed and resuspended at a concentration of 1×10^7^ cells/ml in chemotaxis medium containing: RPMI 1640, 0.5% bovine serum albumin, 25 mM HEPES. A total of 1×10^6^ cells per 100 µl were loaded in the upper transwell chamber (pore size, 5.0 µm; Costar, Corning, NY). Chemokine in 600 µl chemotaxis medium was added to the lower chambers. Upper chambers were transferred on top of lower chambers after a 30 min pre-incubation without chemokine at 37°C in 5% CO_2_ and were incubated for an additional two hours for chemotaxis. The total contents of the lower chambers were collected and equal numbers of counting beads (SPHERO^TM^ AccuCount Fluorescent Particles, Spherotech) were added to each tube according to the manufacturer's instructions. Samples were run on an LSRII flow cytometer (BD Biosciences) and fifty thousand events were acquired to obtain numbers of migrating cells. Analysis was done using FlowJo software (Tree Star).

### Transfection for Expressing CXCR6-YFP Fusion Protein

The sequence encoding CXCR6 was inserted into the pEYFP-N1 vector (Clontech Laboratories, Inc., Palo Alto, CA) to encode a CXCR6 C-terminal fusion protein with an enhanced yellow-green florescent protein (YFP). The plasmid encoding CXCR6-YFP or a GFP control plasmid (pmaxGFP) (Amaxa, Gaithersburg, MD) was introduced into cells using the Nucleofector^®^ Kit V according to the manufacturer's instructions (Amaxa).

### Proliferation and Survival Assays

Twenty-four hours after transfecting cells to express either CXCR6-YFP or GFP, cells were resuspended in 0–5 µg/ml CXCL16 and harvested 48 hours later. For some experiments, cells were loaded with 2 µM Far Red DDAO dye (7-hydroxy-9H (I,3-dichloro-9,9-dimethylacridin-2-one) before culturing and for some experiments harvested cells were stained with Annexin V-PE (BD Biosciences) according to manufacturer's instructions. Samples were analyzed using an LSRII flow cytometer (BD Biosciences) after adding counting beads (SPHERO^TM^ AccuCount Fluorescent Particles, Spherotech) together with 5 µl of Dead Cell Discriminator (CALTAG Laboratories) or propidium iodide. Numbers of live, dead, and in some cases proliferating and non-proliferating cells were determined using FlowJo software (Tree Star).

### Statistical analysis

Data are expressed as the mean +/− SEM. Statistical comparisons of means were performed by unpaired Student's *t* test. For prostate array data, the Kruskal-Wallis or Mann-Whitney tests were used. All statistical tests were two-sided.
